# Moisture Sorption by Low‐Cost Pyridinium‐Based Protic Ionic Liquids: Kinetics and Physico‐Electrochemical Properties

**DOI:** 10.1002/open.202400165

**Published:** 2024-11-26

**Authors:** Sayyar Muhammad, Najia, Zarshad Ali, Samina Aziz, Muhammad Hammad Khan, Maroosh Iqbal, Umair Hassan, Jalal Khan, Asad Ali

**Affiliations:** ^1^ Department of Chemistry Islamia College Peshawar 25120 Peshawar Khyber Pakhtunkhwa Pakistan; ^2^ Energy engineering Division of Energy Science Luleå University of Technology 97187 Luleå Sweden

**Keywords:** Conductivity, Cyclic voltammetry, Ethanol oxidation, Electrocatalysis, Kinetics, Protic ionic liquids, Pyridine, Sustainable chemistry

## Abstract

We report the synthesis of two pyridinium‐based room temperature protic ionic liquids (PILs), pyridinium bisulfate, [HPyr][HSO_4_] and pyridinium sulphate, [HPyr]_2_[SO_4_] and investigation of the kinetics of their water sorption behaviour and its influence on their density, ionic conductivity, and potential windows. The PILs were synthesized by the reaction of pyridine base with an acid, H_2_SO_4_, under solventless conditions, and confirmed by FTIR spectroscopy and 1H NMR spectra. The appearance vibration bands in the 3095–3252 cm^−1^ range for −NH^+^ stretching in the FTIR spectra and a peak at a chemical shift of 8.439 ppm in the 1H‐NMR of the liquids confirm their synthesis as no such bands/peaks can be seen in that of the pure pyridine spectra. The PILs’ hygroscopic nature was examined by exposing them (5 mL each sample with exposed surface area 3.143 cm^2^) to air for varied time intervals at a relative humidity, RH=58±5 % and T=20±5 °C. Coulometric Karl‐Fischer (KF) titration was used to determine how much moisture each PIL sample absorbed at each time interval. The findings reveal that when the PIL was exposed to air for longer periods of time, more moisture was absorbed, and the results correspond well with the pseudo first‐order kinetic model. The densities and conductivities of several samples of the two PILs were examined, and it was discovered that as the percentage water content of the PILs grew, density decreased but conductivities increased. Furthermore, it was discovered that when temperature rose, the conductivity of each of the PILs increased, and the results fit well to the Arrhenius linear equation since the regression coefficient, R^2^, for each of the samples approached the perfect fit value of one. The electrochemical window (EW) data, the mechanism of moisture oxidation within the EWs of each PIL at Pt and Au electrodes, and the electrocatalytic role played by the Pt and Au surface oxides during ethanol oxidation are evaluated and discussed in light of their future sustainable energy applications.

## Graphical abstract

This paper focuses on the synthesis of low‐cost pyridinium‐based protic ionic liquids, [HPyr][HSO_4_] and [HPyr]_2_[SO_4_] and on the study of their physico‐electrochemical behaviour including their hygroscopicity, ionic conductivity, and electrochemical windows. The electro‐oxidation of ethanol is also studied to highlight their potential use in energy applications such as for direct ethanol fuel cells.

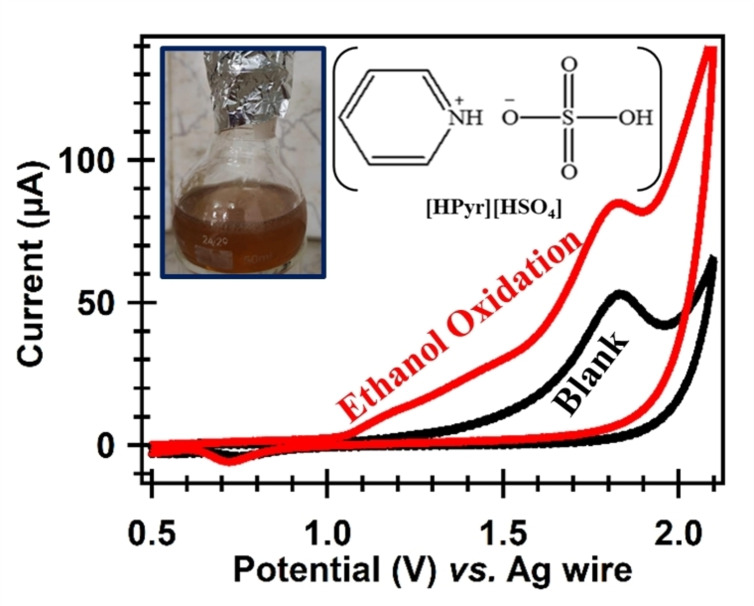

## Introduction

Understanding the physicochemical and electrochemical behaviour of ionic liquids (ILs) is important in relevance to their use in energy conversion and storage devices as well as for newer industrial and commercial applications. Because of the special qualities that ILs possess, there has been a great deal of research conducted over the last three decades regarding the usage of ILs as more ecologically friendly alternatives to conventional organic solvents.^[1]^ ILs are formally defined as liquids that consist entirely of ions usually organic cations and organic or inorganic anions, and which are liquid below 100 °C.^[2]^ Protic ionic liquids (PILs), a subclass of ILs, are those formed by proton transfer from Brønsted acids to Brønsted bases.^[3]^ The physico‐chemical properties of PILs are determined by the presence of hydrogen bonds, labile protons, and proton transfer equilibria.^[4]^ Due to their unique molecular composition and ionic interaction, PILs have relatively low volatility,^[5]^ intrinsically high ionic conductivity,[^6^, ^7^, ^8^] high transport numbers,^[9]^ and great thermal and electrochemical stability,[^10^, ^11^, ^12^] etc. as compared to more volatile organic solvents. Based on these unique properties, PILs have been suggested for their possible use as electrolytes for electrocatalytic reactions related to energy conversion and storage devices such as fuel cells, batteries, supercapacitors, solar cells, or in electrodeposition processes.[^13^, ^14^, ^15^, ^16^, ^17^, ^18^]

However, both water‐miscible as well as water‐immiscible PILs are hygroscopic, i. e. absorb water from atmospheric moisture during the course of their preparation and when exposed to air at ambient laboratory conditions.[^3^, ^10^, ^12^] The hydrophilicity and hygroscopic nature of PILs can be tuned through the choice of cation and anion as it depends upon the nature of the anion and cation of PILs,^[19]^ the time of exposure to the atmosphere, and also on the moisture contents (relative humidity) of the atmosphere.[^3^, ^20^] For example, Chen *et al*.^[21]^ has shown that diethyl‐ammonium formate (i. e., [DEA][FO]) is more hygroscopic, absorbing up to 25 % g/g water from the air in 24 hours at an average temperature of 30 °C and a relative humidity of 57 % as compared to the other nine PILs reported in this study. Speib *et al*.^[19]^ have reported that the water sorption capacity of [BMIm][OAc] ranged from 2100 mg/g for 6.67 mg of sample to 900 mg/g for 64.14 mg of IL sample. The moisture sorption data of the ILs can be fitted to different models or ways to correlate or predict their hygroscopicity. We reported previously the moisture sorption kinetics of four PILs and showed a pseudo‐1st order kinetics model fitted well to the data at a relative humidity of 36±5 % and at 31±5 °C.^[3]^ Arellano *et al*.^[22]^ investigated the moisture sorption kinetics of [BMIM][Br] and [OMIM][Br], and found that the Henderson‐Pabis model fit the data well at 358 K and 85 % RH. After studying the hygroscopicity of anhydrous ILs, Francesco *et al*.^[23]^ used an exponential model to correlate the water sorption capacity with time, whereas Cao *et al*.^[24]^ reported a modified two‐step (mod. two‐step) water sorption process for 18 ILs.

The presence of water in contact with ILs may be useful in some applications, but not in others. For example, the presence of water in humidity sensors or drying agents is desirable, or perhaps required, for other applications (e. g., fuel cells). Lithium batteries, electrochemical supercapacitors, solar panels, electrosynthesis, and field‐effect double‐layer transistors are examples of electrochemical applications that need virtually anhydrous electrolytes. PILs’ hygroscopicity can have a significant impact on their physical properties such as viscosity, electrical conductivity, thermal degradation, and hydrolytic decomposition, variation in reactivity and solvating ability, and electrochemical window.[^3^, ^20^] In short, the aspects governing the macroscopic properties of PILs need to be well‐defined, in particular, measuring properties such as hygroscopic behaviour, thermal stability, density, viscosity, conductivity, and electrochemical behaviour in order to promote their practical industrial applications as electrolytes as well as predict their properties and performance. For this purpose, alkyl ammonium, alkanol ammonium, imidazolium, phosphonium, etc. cations‐based PILs are extensively studied, but not much of the study is devoted to exploring the water sorption property, physicochemical and electrochemical behaviours of pyridinium−based PILs. For example, Duan *et al*.^[25]^ investigated the catalytic activity of three PILs based on 2‐methylpyridinium for tert‐butylation of phenol and the esterification of cyclic olefins with acetic acid. Bandres *et al*.^[26]^ studied the conductivity behaviour as a function of temperature of 1‐butylpyridinium‐based ionic liquids with different anions, Mayrand‐Provencher *et al*.^[27]^ showed that different colored impurities in the PILs based on 2‐methylpyridine (2‐MPy) and trifluoroacetic acid (TFA) can affect both the electrochemical behaviour (on Pt and on GC) but also on the physicochemical properties like conductivity, density, and viscosity, McCune *et al*.^[28]^ evaluated the liquid structure of pyridine−acetic acid mixtures using neutron scattering at various mole fractions of acetic acid, and compared to the structures of neat pyridine and acetic acid, and Rajabi *et al*.^[29]^ examined the synergetic effect of pyridinium trifluoroacetate salts (PMO−Py‐IL) in the sustainable biodiesel‐like esters production. Although, McGrogan *et al*.^[30]^ used pyridinium hydrogen sulphate, [HPy][HSO_4_] in combination with pure H_2_SO_4_ and studied their H‐bond interaction, they did not report about its hygroscopicity, conductivity, and electrochemical properties.

**Scheme 1:**
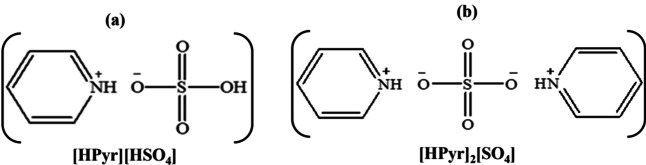
Structure formula of the synthesized PILs.

In this regard, we synthesized and characterized low‐cost pyridinium bisulphate, [HPyr][HSO_4_] and pyridinium sulphate, [HPyr]_2_[SO_4_], PILs (shown in Scheme 1) and investigated their water sorption ability (hygroscopicity) from the moisture atmosphere under ambient laboratory conditions, its kinetics, mechanism, and studied the impact of the moisture‐laden‐PILs on their conductivity and electrochemical behaviour. Finally, we have investigated the electrocatalytic behaviour of these PILs toward ethanol (EtOH) oxidation in the medium. One of the PILs with structure (b) in Scheme 1, is also commercially available with the name pyridine sulfate having other synonyms as pyridinium hydrogensulfate; pyridine hemisulfate; pyridine sulfate (1 : 1); pyridinium hydrogen sulphate having CAS no. 543‐54‐4.^[31]^

## Materials and Methods

### Chemicals and Apparatus

The chemicals sulphuric acid, H_2_SO_4_ (95–98 %) and pyridine, C_5_H_5_N: (≥95 %) used for the synthesis of ionic liquids were purchased from trusted chemical suppliers Merck MRK Germany and Analar BDH Chemicals, England. 0.5 % H_2_O and 0.02 % of non‐volatile matter were present as impurities in pyridine. Sulphuric acid contained <0.0001 % NO_3_^−^, <0.00001 % Cl^−^, and As ions, <0.0005 % each of NH^+^ and alloy Pb, <0.001 % Pb, and <0.0002 % Fe ions. All the chemicals were used as received without any further purification or special separation. The PIL synthesis equipment had a three‐necked flask, a reflux condenser, an ice bath, a separating funnel that served as an acid dropper, and a heating plate/magnetic stirrer.

### PILs Synthesis

**Scheme 2:**
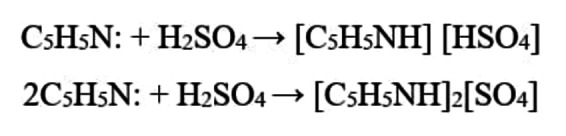
Reaction showing the PILs formation.

Pyridine (42.4 mL) and H_2_SO_4_ (27.5 mL) were reacted in a 1 : 1 ratio to produce [HPyr][HSO_4_], whereas for [HPyr]_2_[SO_4_] preparation, 42.4 mL Pyridine and 13.7 mL H_2_SO_4_ were reacted in 2 : 1 in a 3‐necked flask at ambient temperature under solventless circumstances using a conventional technique.^[32]^ As these are exothermic processes, a thermometer was placed in one of the flask's necks to measure the temperature rise and control it to room temperature. A condenser was placed in the flask's central neck to condense any vapors produced by the reacting mixture. A dropping funnel containing the acid was fitted in the 3^rd^ neck of the 3‐neck flask. A dropping funnel was used to add the acid to the base of a three‐necked flask. After adding the acid in drips, the mixture was progressively heated to 80 °C and stirred for 24 hours. After cooling to room temperature, brown‐colored and viscous liquids were formed. The reaction Scheme 2 depicts the reaction between pyridine and H_2_SO_4_ in 1 : 1 for the synthesis of pyridinium bisulphate and 2 : 1 for the formation of pyridinium sulphate;

### Characterization of the PILs

The transfer of protons from the studied acid to the base during the synthesis of the PILs was confirmed through Fourier transform infrared, FTIR spectroscopy. The FT‐IR transmission spectra of the PILs were taken on a Perkin Elmer (UK) FTIR spectrometer in the interval 4000–400 cm^−1^. To obtain the spectra, 2 mL each of [HPyr][HSO_4_] and [HPyr]_2_[SO_4_] were mixed with 20 mg KBr to make its paste and fabricated onto pallets with a diameter of approximately 13 mm. The molecular structure of ILs was also confirmed by 1H NMR using a Bruker 300 MHz NMR instrument Perkin Elmer (UK) in deuterated chloroform (CDCl_3_). The moisture contents of the various PIL samples were determined by using the Karl‐Fischer Volumetric Titrator – HI903 from Hannas Instruments with accuracy ±0.5 % (relative) and ±0.2 % (absolute) and resolution ranging from 1 ppm to 0.0001 %. For conductivity measurements, Jenway model 4510 conductivity meter probe was used. The electrochemical characterization (cyclic voltammograms, CVs) of the PIL samples was carried out using a computer‐controlled Autolab Potentiostat (Netherlands).

### Moisture Uptake Analysis

The presence of water in the ILs alters the ionic interactions between the ions as well as their physical characteristics, including their solvating power, electrical conductivity, viscosity, and reactivity. Practically speaking, it is very helpful to know how much water an ionic liquid can absorb from its surroundings because the use of these liquids for different electrochemical purposes, requires an understanding of their moisture content values.[^28^, ^33^] For this purpose, equal volumes (5 mL) of the [HPyr][HSO_4_] and [HPyr]_2_[SO_4_] were taken in small glass vials exposed to air at 20±5 °C and 58±5 % relative humidity for varying durations of time (24, 48, 72, 96, 120, 148, 172, 196, and 216 hours) in order to ascertain their moisture uptake ability. The surface area of the PILs exposed to the air moisture was 3.143 cm^2^. The Hanna titrator was then used to ascertain the water content of each of these samples. Pseudo first order kinetic model was applied to the moisture contents data of the PILs using Equation 1.

(1) $$\;ln{\%\left[H_{2}O\right]}_{t}=\;-kt+\ln\%{\left[H_{2}O\right]}_{o}$$

In the equation, % [H_2_O]_o_ represents the initial water fraction of the benchtop PIL samples, 0.765 % in [HPyr][HSO_4_] and 0.661 % in [HPyr]_2_[SO_4_] at t_0_. Where % [H_2_O]_t_ represents the percent fraction of each of these PIL samples for each of the exposed time intervals, t. K is the rate constant, which can be found from the slope of the plot.

The density of [HPyr][HSO_4_] and [HPyr]_2_[SO_4_] was calculated using a straightforward and standard approach. For this reason, a one millilitre syringe was cleaned, dried, and weighed using an analytical balance. A predetermined volume of each PIL sample (at t_0_) was drawn into the syringe, and the full syringe was weighed. Equation (2) determined the density of the sample based on its mass and volume.

(2) $$density=\frac{\left(mass\;of\;filled\;syringe\right)-\left(mass\;of\;empty\;syringe\right)}{\left(volume\;of\;PyrHS\right)}$$

To improve accuracy, the same procedure was done three times for each PIL sample, followed by the calculation of an average density. A similar approach was used for the remaining moist samples of the two PILs.

### Conductivity Analysis

The conductivity of each of the PIL samples was determined at various temperatures (298, 313, 323, 333, 343, 353, 363, 373, 383, and 393 K). For this purpose, a freshly prepared 0.1 M KCl solution was used for its calibration at a cell constant of 1.0 cm. The probe was then immersed in the PILs sample and their conductivity (in millisiemens per centimetre, mS.cm^−1^) was determined at various temperatures with an error bar ±0.5 %. The acquired results were plotted and analyzed. The Arrhenius equation (Equation (3)) was used to fit the data showing how temperature influenced the conductivities of the PILs sample.

(3) $$ln\sigma =ln\sigma^{o}-\frac{E_{a}}{R\;T}$$

The conductivity symbol is σ, the Arrhenius factor is σ°, Ea is the process's activation energy as determined by the slope of the plot, and T is the temperature in Kelvin (K).

### Electrochemical Analysis

A 3‐necked glass cell containing a working electrode (either Pt or Au), a counter electrode (Pt‐mesh), and an Ag quasi‐reference electrode was used for electrochemical characterization of the PILs. The working electrode was polished with an aqueous alumina suspension before each CV was recorded. It was then rinsed, ultrasonically cleaned in deionised water, and dried under a N2 stream. The electrochemical cell was charged with around 5 mL of the PIL in order to record CVs, and after 20 minutes, N_2_ gas was evacuated to eliminate any remaining dissolved O_2_. The Window CVs and CVs for ethanol oxidation in the PILs were taken by adopting the procedure described in our previous articles.[^10^, ^11^]

## Results and Discussion

Following the procedure outlined in the experimental section, pure [HPyr][HSO_4_] (0.765 % of H_2_O) and [HPyr]_2_[SO_4_] (0.661 % of H_2_O) were produced having brownish colour. The PILs were prepared under ambient laboratory conditions and were not heated further. This avoids the common problem of thermal decomposition by avoiding the need for purification methods that require heating the PILs. The ^1^H NMR spectra (**Supporting Information, Figure** **S1**) show no impurities were present in the PILs.

### Characterization by FT‐IR Spectroscopy

**Figure 1:**
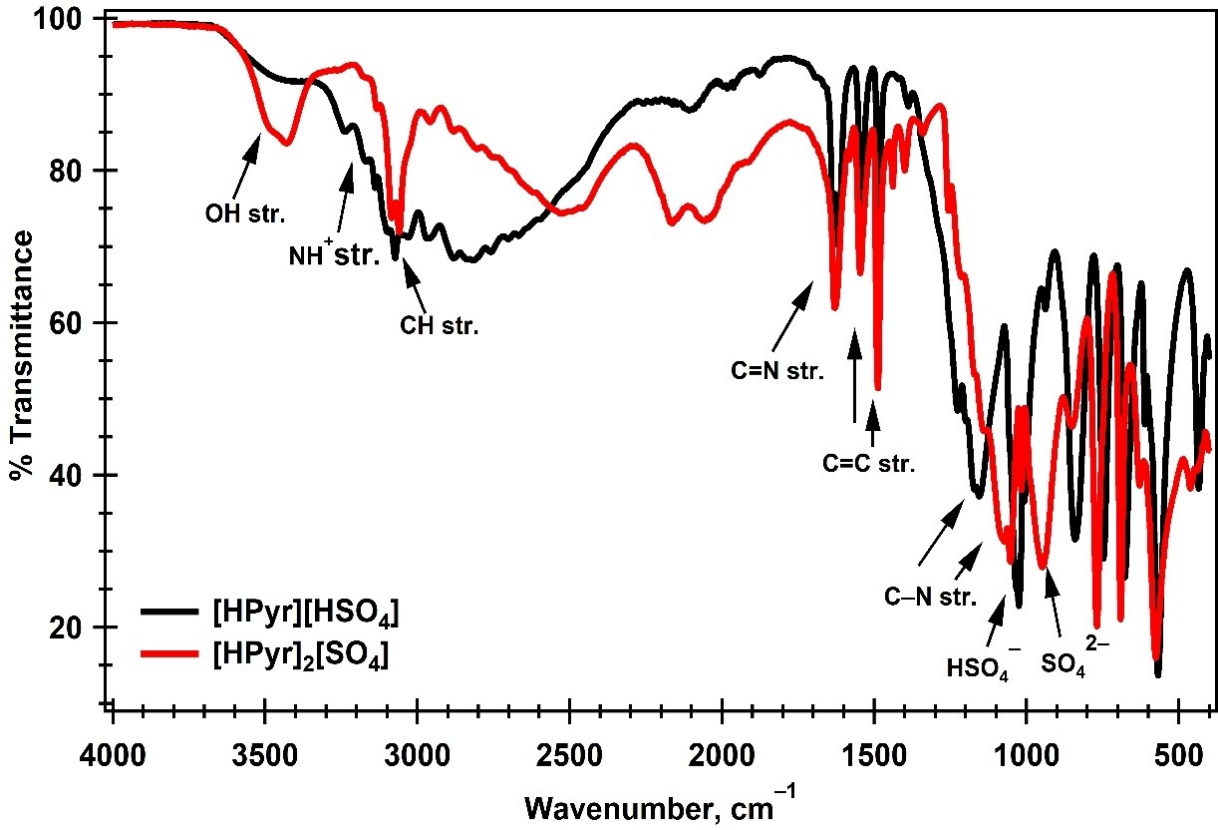
FTIR spectra of [HPyr][HSO_4_] and [HPyr]_2_[SO_4_].

The transmission *vs*. wavenumber (cm^−1^) plot of the FT‐IR spectra of [HPyr][HSO_4_] and [HPyr]_2_[SO_4_], obtained in the 400–4000 cm^−1^ range, is shown in Figure 1. The figure indicates that the vibration bands observed in the FT‐IR spectra of [HPyr][HSO_4_] at 3228 cm^−1^, 3147 cm^−1^, and 3092 cm^−1^ and in the FT‐IR spectra of [HPyr]_2_[SO_4_] at 3243 cm^−1^, 3146 cm^−1^, and 3113 cm^−1^ are exclusively ascribed to −NH^+^ stretching vibrations. Additionally, the FT‐IR spectra of [HPyr][HSO_4_] and [HPyr]_2_[SO_4_] show peaks at 3446 cm^−1^ and 3429 cm^−1^, respectively, which correspond to −OH stretching vibration. The FT‐IR spectra of pure pyridine do not contain any of these two vibration bands.^[34]^ The stretching vibrations obtained are at 1026 cm^−1^ in the FT‐IR of [HPyr][HSO_4_] corresponds to bisulphate anion and that at 947 cm^−1^ and 850 cm^−1^ in the FT‐IR spectra of [HPyr]_2_[SO_4_] correspond to sulphate anion. The pyridine characteristic peaks for CH, C=N, C=C, and CN are also evident in the FT‐IR spectra of [HPyr][HSO_4_] and [HPyr]_2_[SO_4_].

### Hygroscopicity Analysis of the PILs

As mentioned, PILs are essentially hygroscopic due to their ionic structure, which can have significant effects on their physicochemical and electrochemical behaviour, therefore, their concentration must be measured. In this regard, we have measured the moisture contents of the bench‐top samples of our synthesized PILs through coulometric Karl‐Fischer titration and found 0.765 % water in [HPyr][HSO_4_] and 0.661 % in [HPyr]_2_[SO_4_]. The initial water fractions in the two PILs are caused by water impurities in the precursor reagents (pyridine, 95 % and H_2_SO_4_, 95–98 % pure), as well as moisture absorbed from the atmosphere throughout the experimental procedure for their synthesis. When the PIL samples were exposed to moist air for varying lengths of time at RH=58±5 % and T=20±5 °C, as described in the experimental section, their water content increased as a result of the absorption of moisture from air. However, as compared to the literature report on water uptake by other PILs,[^3^, ^20^] the water uptake by the PILs, [HPyr][HSO_4_] and [HPyr]_2_[SO_4_] was low. The low water uptake here can be attributed to; 1) the exposed surface area of PILs was very small (3.143 cm^2^) as a 5 mL sample was taken in a 10 mL glass vial, 2) The cation of the ionic liquid makes interaction or H‐bonding with the incoming water molecules, and here the cation is a nonpolar, planar pyridine, which is a hydrophobic site, so apart from H‐bonding at the N−H bond site, no other site of pyridinium cation can form H‐bond interaction with water. The findings from Karl‐Fischer titration, shown in Table 1 **(Supporting Information)**, demonstrate the hygroscopic nature of these PILs.

**Figure 2:**
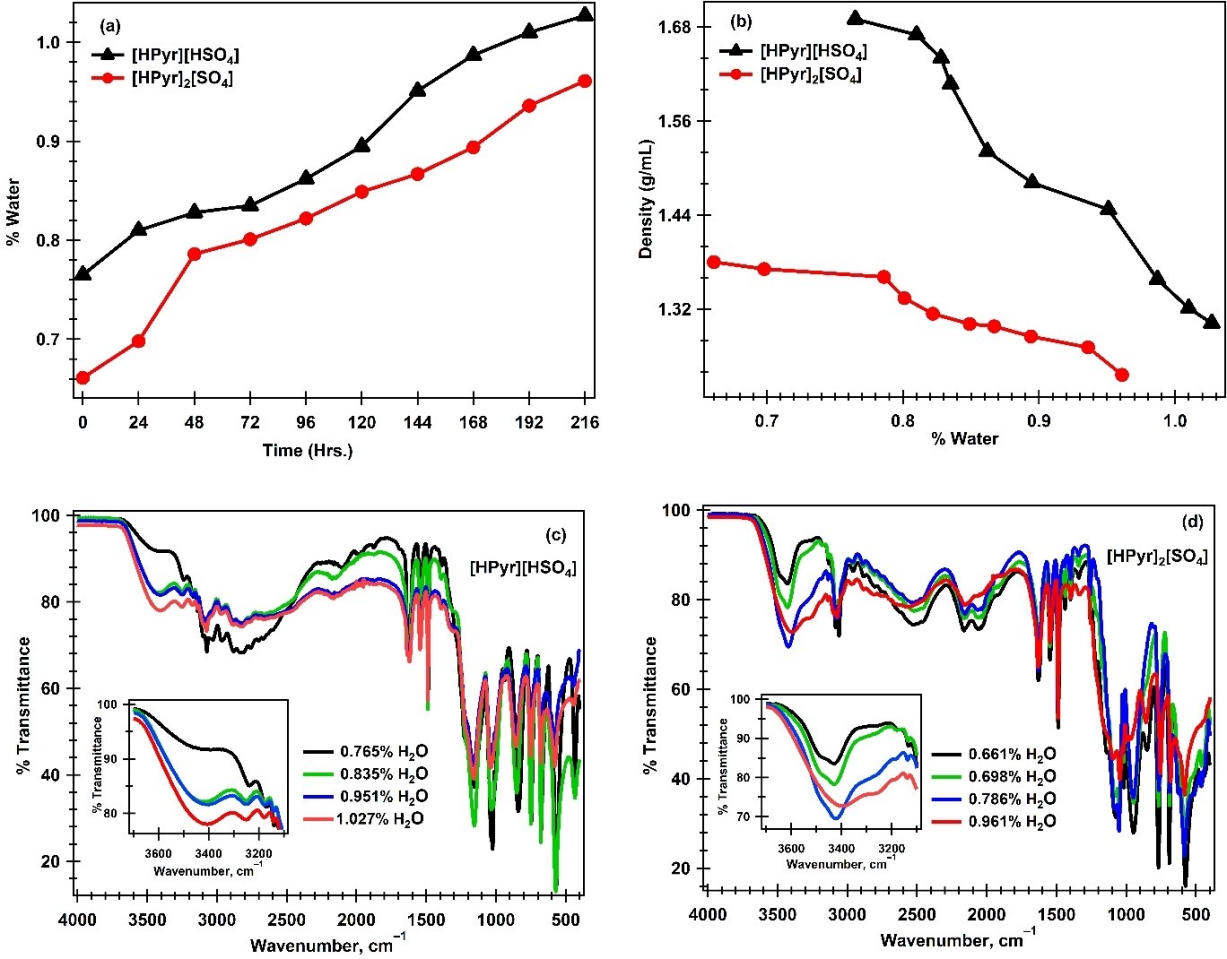
plot of (a) moisture contents of the PILs vs. time of exposure to air at T=20±5 °C and RH=58±5 %, (b) density vs. moisture contents of the PILs and effect of moisture contents on the FTIR spectra (c) of the [HPyr][HSO_4_] and (d) of the [HPyr]_2_[SO_4_].

Figure 2(a) depicts a graph of the percentage of water absorbed by the PILs across various time periods for each of the air‐exposed samples. The plot shows that the samples of each PIL that were exposed to air for a longer period of time under ambient laboratory settings absorbed more moisture than those that were not exposed to air. It shows that both [HPyr][HSO_4_] and [HPyr]_2_[SO_4_] show a very strong hygroscopic nature. It is due to the formation of a hydrogen bond by the air moisture with the pyridinium cation and bisulphate and sulphate anions of the two PILs, respectively.[^26^, ^30^] In comparison to our previous studies, the benchtop sample of [HPyr][HSO_4_] contains a higher percentage of water (0.765 %) than that of the triethylammonium bisulphate, TEAHS (0.410 %) while less than that of the benchtop sample of triethanolammonium bisulphate, TEOAHS (1.01 %). The % water fraction of the [HPyr][HSO_4_] reached 1.01 % (TEOAHS value) only when its sample was exposed to air for 196 hours.^[3]^ This shows that pyridinium‐cation forms more hydrogen bonding with water to capture more moisture from air than that of triethylammonium cation but forms less hydrogen bonding with water from air as compared to triethanolammonium cation. This means that [HPyr][HSO_4_] is less hygroscopic as compared to TEOAHS and more than TEAHS.

Because of its importance in related applications, density is the most commonly measured and reported physical property of ILs. In general, a substance's density is influenced by temperature, pressure, relative molecular mass, molecule interactions, and molecular structure.^[35]^ Figure 2(b) depicts the density of [HPyr][HSO_4_] and [HPyr]_2_[SO_4_] in g mL^−1^ versus their percentage water contents. The figure depicts how the density of each PIL reduced as the water content of each sample increased.^[36]^ The presence of water impurity reduces the densities of the PILs since water has lower densities than the PILs under investigation. This is because, as the moisture intake of the PILs rises, the volume increases while the density drops.^[37]^

Evidence for the presence of hydrogen bonding can be obtained from IR spectroscopy. In order to explore this, we have taken the FTIR spectra of various wet samples of the PILs containing variable amounts of moisture, which are shown in Figure 2 (c) and (d). It is clear from the figure that the −OH stretching band of the benchtop samples of [HPyr][HSO_4_] and [HPyr]_2_[SO_4_], black plot, is weak in absorbance and broad in the wavenumber range ∼3700 to∼3100 cm^−1^. As the water content of the samples increased, so did the depth and breadth of the vibrational bands in each case, as shown in the inside figures of the Figures 2(c) and (d). As a result, water makes up the majority of the −OH stretching band in hydrated PILs. Thus the −OH stretching band is mostly caused by the stretching vibrations of water molecules. The −OH stretching band is believed to be sensitive to variations in the hydrogen‐bonding network of water molecules. This also confirms the production of ILs and their moisture absorption capabilities. A similar kind of result is reported by Inbaraj *et al*.^[38]^ in which they studied the effect of water contents on the −OH stretching bands of the cholinium dihydrogen phosphate, [Ch][dHp] IL.

**Figure 3:**
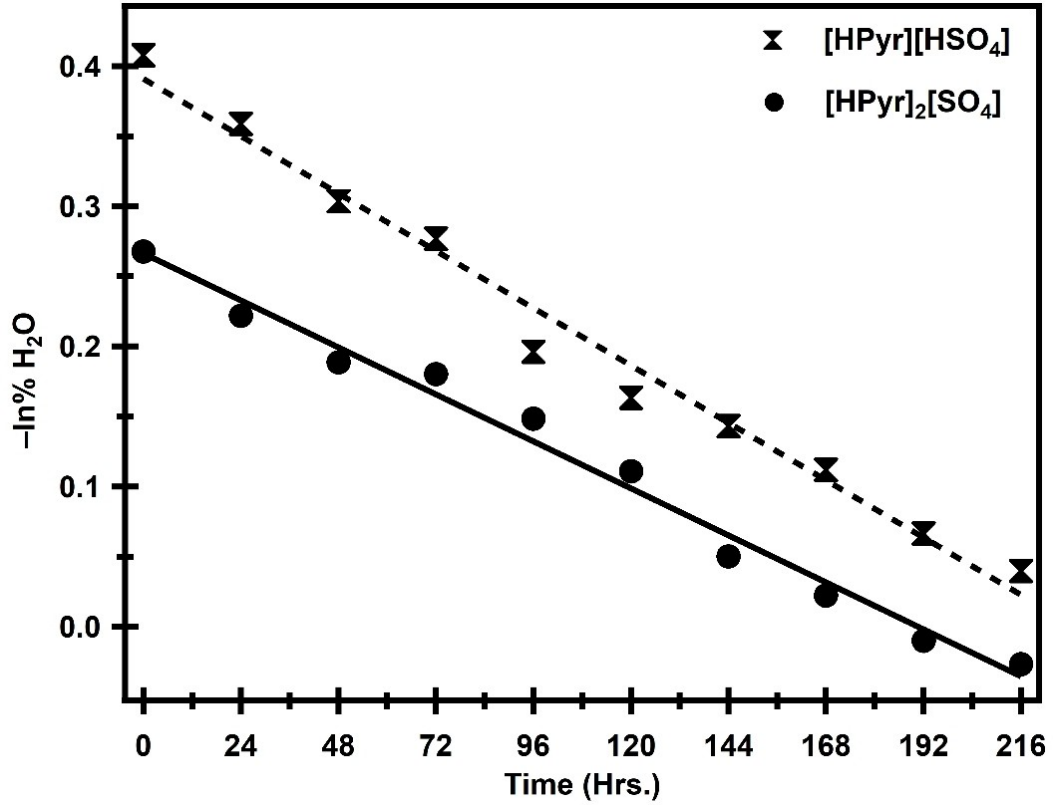
plot of fitting the data to a pseudo first order kinetic model as −ln% [H_2_O] vs. time for various samples of [HPyr][HSO_4_] and [HPyr]_2_[SO_4_].

Figure 3 shows a plot of −ln% [H_2_O] *vs*. time of air exposure to validate the results for pseudo‐first‐order kinetics (Equation (2)). The graphic shows a linear graph for both liquids, [HPyr][HSO_4_] and [HPyr]_2_[SO_4_] in accordance with the reported literature.^[3]^ The correlation coefficient, R^2^, obtained from the straight‐line plot for [HPyr][HSO_4_] is 0.912, and for [HPyr]_2_[SO_4_], it is 0.966, showing that the data corresponds well statistically to first order kinetics because the values are closer to the ideal R^2^.

### Conductivity Analysis

**Figure 4:**
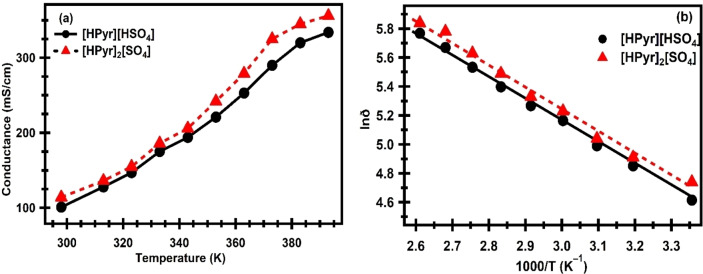
Plots of (a) conductivity vs. temperature and (b) ln σ vs. 1000/T for [HPyr][HSO_4_] and [HPyr]_2_[SO_4_].

The conductivities of the two PIL were measured across a temperature range of 298 K to 393 K. The data from Table 2 **(Supporting Information)** is shown as conductivity *vs*. temperature for both [HPyr][HSO_4_] and [HPyr]_2_[SO_4_] and plotted as shown in Figure 4 (a). The figure clearly indicates that conductivity increases linearly with temperature. One factor contributing to increased conductivity is that when temperature rises, the kinetic energy of the cations and anions increases, and the interaction between the ions in PILs reduces, increasing the mobility of the ions towards the electrode probe. The conductivity data for several samples of [HPyr][HSO4] and [HPyr]2[SO4] at different temperatures was plotted as a logarithm of conductivities against the reciprocal of temperatures (Table 2 Supporting Information) using the linear form of the Arrhenius Equation (5).

Figure 4 (b) shows the relationship between ionic conductivity and inverse temperature. The log σ *vs*. 1000/T plot of [HPyr][HSO_4_] and [HPyr]_2_[SO_4_] samples is linear. In all of these cases, the regression coefficients and R^2^ values for each sample are close to the ideal value of R^2^=1 (R^2^=0.9975 for [HPyr][HSO_4_] and R^2^=0.9934 for [HPyr]_2_[SO_4_]), indicating a good statistical fit for the Arrhenius linear equation. The Ea computed from the slope of the Arrhenius equation for [HPyr][HSO_4_] and [HPyr]_2_[SO_4_] samples were 12.42 kJ/mol and 12.62 kJ/mol, respectively. The Ea value for [HPyr][HSO_4_] is lower than that of [HPyr]_2_[SO_4_], showing that this liquid is less viscous since it contains more water (0.765 %) than [HPyr]_2_[SO_4_] (0.661 %). This shows that the conductivity of [HPyr][HSO_4_] increases quicker with temperature than that of [HPyr]_2_[SO_4_].

### Electrochemical Characterization of the PILs

**Figure 5:**
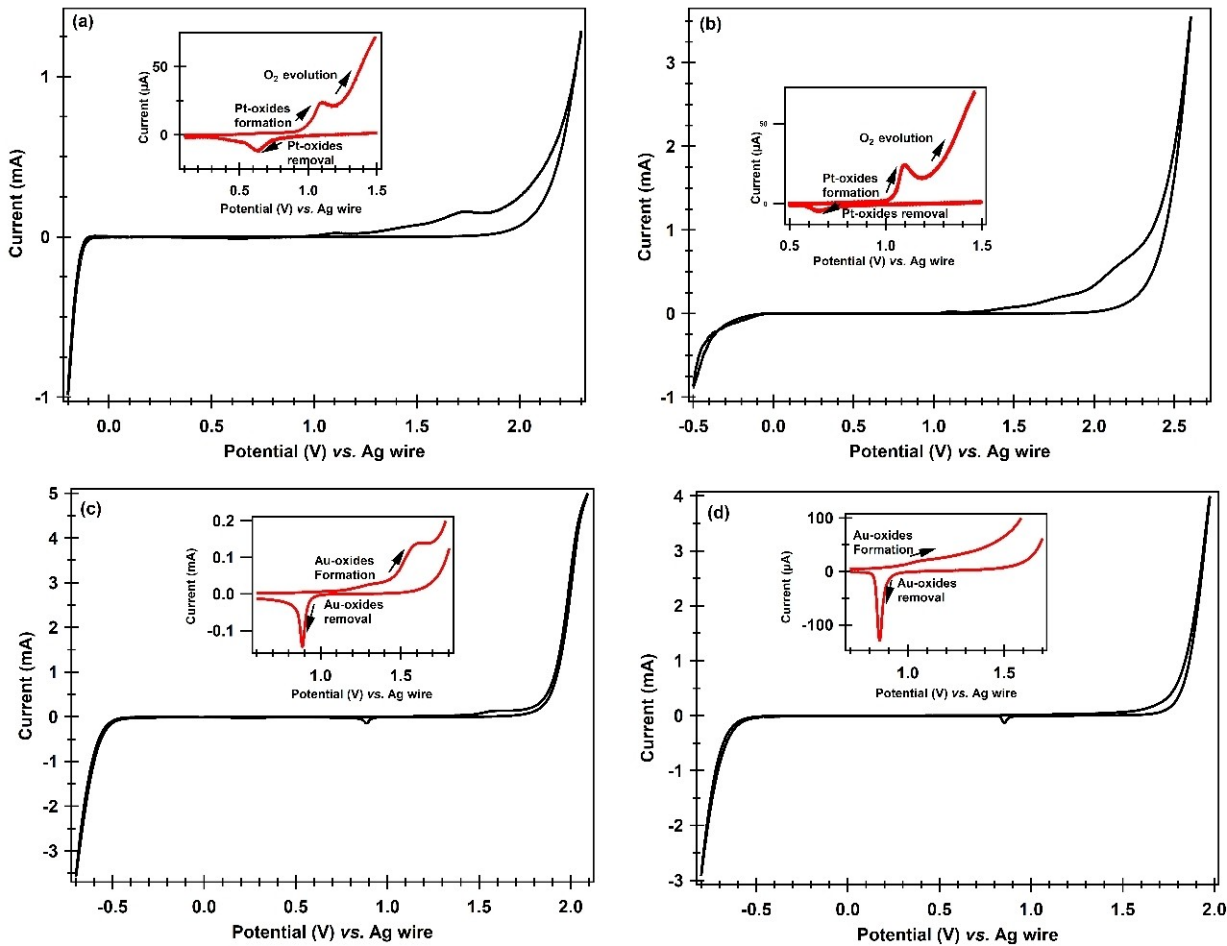
CVs showing the EWs in [HPyr][HSO_4_] at (a) Pt and (c) Au and in [HPyr]_2_[SO_4_] at (b) Pt and (d) Au vs. Ag wire quasi reference electrode at a scan rate of 50 mV/s recorded at 303 K. **Inset**: show CVs in the potential range where surface oxides formation takes place at Pt and Au due to water oxidation.

**Scheme 3:**
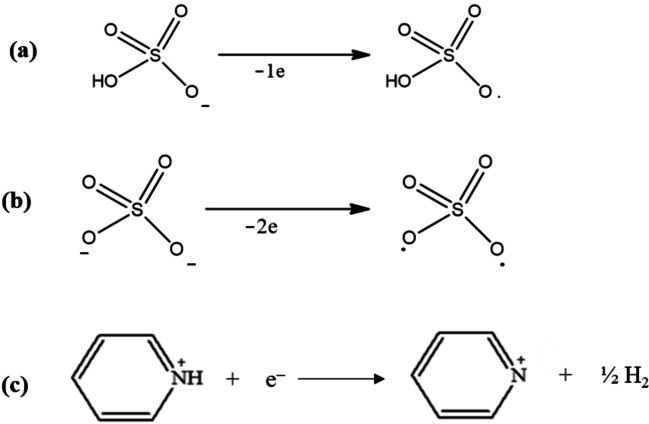
Showing the oxidation of the anions (a) [HSO_4_]^−^ and (b) [SO_4_]^2−^ and (c) reduction of the [HPyr]^+^ cation.

The electrochemical window (EW) of a liquid is an important parameter in several domains, including electrochemistry and energy storage. Therefore, understanding and optimization of the electrochemical window is an important parameter to be determined for a neoteric liquid. EW refers to the range of potentials over which a liquid electrolyte can remain stable without suffering undesired reactions like electrolysis or breakdown. The EWs of the synthesized PILs were determined by cyclic voltammetry. CVs were recorded at Pt and Au as working electrodes with Ag wire serving as a quasi‐reference electrode at 303 K and a scan rate of 50 mV/s as shown in Figure 5 (in [HPyr][HSO_4_] at Pt (a) and Au (c) and in [HPyr]_2_[SO_4_] at Pt (b) and Au (d)). The anodic and cathodic limits and the overall EWs (= anodic potential limit – cathodic potential limits) were determined at each of the electrodes for each of the PILs and are summarized in Table 3 (**Supporting Information**). The oxidation potential limit was determined from the steep rise in current at ~2.0 V in the positive going scan in each of the PIL, reaching a limiting value. This corresponds to the oxidation of anions, specifically [HSO_4_]^−^ and [SO_4_]^2−^ (as indicated in Scheme 3 below). From this point forward, the potential was inverted, resulting in a sharp increase in cathodic peak current. From this, the reduction potential limit was attained, which is associated with the reduction of pyridinium cation, [HPyr]^+^ (as shown below in the reaction Scheme 3), where, the [HPyr]^+^ was reduced to pyridine and hydrogen during the negative going sweep.

It can be seen from Figure 5 as well as from the data in Table 3 **(Supporting Information)** that the EW in [HPyr][HSO_4_] and [HPyr]_2_[SO_4_] at Pt electrode were 2.1 V and 2.3 V, respectively. Similarly, the EW at Au electrode was 2.3 V and 2.4 V in [HPyr][HSO_4_] and [HPyr]_2_[SO_4_], respectively. So, by comparing the EW in each of the PIL, the window at the Au electrode is wider than that at Pt,[^3^, ^14^, ^15^, ^16^] showing that oxidation of cations and reduction of the pyridinium cation take place earlier at a Pt electrode as compared to that at the Au electrode. The image shows that each PIL has a distinct EW at each electrode, demonstrating that EW is dependent on the electrode material in these liquids as well as the type of the liquid's cations and anion.

The inset of each figure shows CVs in the potential range where a tiny oxidative wave is seen during the forward or positive going potential scan at ~1.0 V at Pt and ~1.3 V at the Au electrode. These oxidative waves in both of the PILs are attributed to the formation of surface oxide at Pt (PtOH/PtO/PtO_2_) and at an Au (Au_2_O/Au_2_O_3_) due to oxidation of the omnipresent and adventitious water contents 0.765 % in [HPyr][HSO_4_] and 0.661 %, in [HPyr]_2_[SO_4_].[^14^, ^39^]

It is observed that with the increase in water contents of PILs, their EW decreases and oxide layer thickness increases, as we have reported in our previous study in other PILs.[^3^, ^10^, ^11^, ^14^] High temperature study of these liquids is important in order to use these neoteric electrolytes in the future intermediate temperature fuel cells. Therefore, we have reported in our previous studies the effect of temperature on the EW of trialkyl ammonium‐based[^3^, ^10^, ^11^, ^14^, ^39^] PILs, and here in we have also investigated this effect in the two Pyridinium‐based PILs, [HPyr][HSO_4_] and [HPyr]_2_[SO_4_], and the CVs obtained (**Figure** **S2 in the supporting information**) show similar results as we have reported previously. That is, as the temperature increases, both the oxidation of anion of the PILs and reduction of the cation [HPyr]^+^ occur at lower potential. This shows that with the increase in temperature, the cation and anion of the PILs diffuse quickly towards the electrode surface for oxidative and reductive decompositions. In addition, due to the increase in temperature, the rate and amounts of formation of the surface oxides/hydroxides at the Pt and Au electrode surfaces and their corresponding reduction during the reverse going sweep also increase as evident from the figures shown in the inset of each of the figures (**Figure** **S2**).

**Figure 6:**
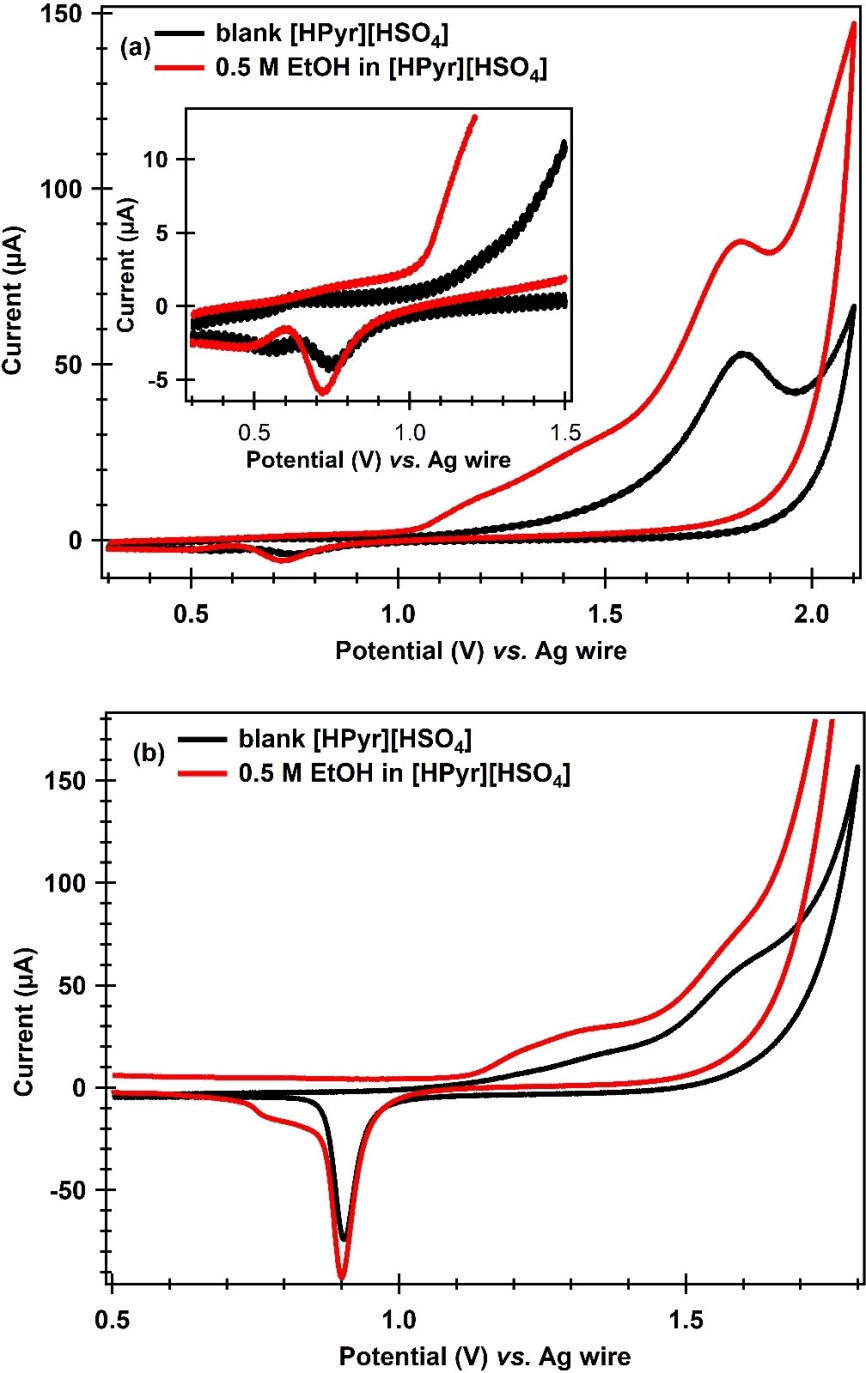
CV obtained at a scan rate of 50 mV/s in [HPyr][HSO_4_] containing 0.5 M ethanol at (a) Pt and (b) Au electrodes vs. Ag wire quasi‐reference electrode at 298 K.

A study of the ethanol oxidation in these proton conducting electrolytes can play a crucial role in enabling the efficient operation of intermediate temperature fuel cells by enhancing performance, reducing blockage of Pt catalyst deactivation due to early oxide formation on its surface in aqueous electrolytes that causes sluggish oxygen reduction reaction (ORR), improving stability, and promoting environmental sustainability by avoiding the use of H_2_ fuel in fuel cells, which has safety and transportation issues. In this regard, we also studied the electrochemical behaviour of Au and Pt electrodes during ethanol oxidation in [HPyr][HSO_4_] electrolytes as shown in Figure 6. Figure 6(a) shows the cyclic voltammograms of the Pt electrode in N_2_ saturated 0.5 M EtOH in [HPyr][HSO_4_] at 298 K and Figure 6 (b) at an Au electrode recorded at a scan rate of 50 mV/s. The black lines in each of these Figures (a) and (b) show the CVs recorded in N_2_ saturated [HPyr][HSO_4_] without ethanol. As described earlier, in the blank CVs, an oxidative wave appeared slightly above 1.0 V at Pt (Figure 6 a) after the capacitive current from 0.3 V up to 1.0 V and above 1.2 V at Au (Figure 6 b) after the capacitive current from 0.5 V up to 1.2 V, corresponding to the oxide formation on these electrodes, respectively. When CVs were recorded at both of these electrodes in the 0.5 M of ethanol solution in [HPyr][HSO_4_], as shown by red lines in each of these figures (a) and (b), a wider oxidative wave having high current was observed after 1.0 V (Figure 6 a) at Pt catalyst and after 1.2 V (Figure 6 b) at Au catalyst that coincides with the onset of surface oxidation in blank [HPyr][HSO_4_] at each of these electrodes. This oxidation wave compared to the blank CVs in each of these figures (a) and (b) is thought to be completely due to EtOH oxidation in the PILs.^[14]^

The surface Pt‐oxide/hydroxide (PtOH/PtO) and Au‐oxides (Au_2_O and Au_2_O_3_) adsorbed species are reduced from the surface during the reverse sweep below 0.9 V at the Pt catalyst with a peak current at 0.75 V and below 1.0 V at the Au catalyst with a peak current at 0.9 V in both the blank CVs (blank line) as well as in EtOH containing CVs (red line). In the case of EtOH containing [HPyr][HSO_4_], once the adsorbed oxides strip off the Pt surface, a small oxidative wave appeared just below 0.75 V, which is also due to EtOH oxidation. No such oxidation is seen at the Au electrode after its surface gets free from the Au‐oxides, showing that Pt is a good catalyst for EtOH oxidation in [HPyr][HSO_4_]. However, compared to 0.5 M aqueous H_2_SO_4_,^[14]^ the poor current response could be attributed to the high viscosity of [HPyr][HSO_4_] and the reduced mass transit of EtOH in the PIL. Another cause could be a lack of water coverage on the Pt surface in the PIL, as water is required as a precursor to the production of Pt−OH and Pt−O, which are then required to oxidize EtOH. This work demonstrates that [HPyr][HSO_4_] can be utilized as an electrolyte to create direct ethanol fuel cells (DEFCs) capable of operating at higher temperatures that at which H_2_/O_2_ fuel cells operate (below 80 °C) due to dehydration of the Nafion membrane that can affect the performance of H_2_/O_2_ FCs.

## Conclusions

Two new low‐cost PILs, namely [HPyr][HSO_4_]) and [HPyr]_2_[SO_4_], were successfully synthesized as confirmed by their FT‐IR and ^1^H‐NMR spectra. The water sorption capacity of both PILs, along with their sorption kinetics, was also studied. It was found that increasing the time interval for PIL exposure to air causes more moisture uptake. This sorption process follows the pseudo‐first order kinetic equation, as the R^2^ value obtained in each case was close to the ideal statistical fitting value. The increase in width and depth of the absorption bands in 3300–3600 cm^−1^ in the FTIR spectra, corresponding to PIL samples with higher water content, further confirmed their hygroscopic nature. Notably, the conductivity of both PILs rises linearly with increasing water content and the temperature of medium. A satisfactory numerical fit was also obtained using the Arrhenius linear equation, based on conductivity data acquired at various temperatures. Electrochemical investigation revealed that in each EW of the PILs, a wider EW was found at the Au electrode than at the Pt electrode. Furthermore, during positive potential scans, water oxidation at Pt and Au electrodes in the PILs resulted in the formation of oxides on each electrode surface, which played an electrocatalytic role during ethanol electro‐oxidation. The PILs are proposed for use in intermediate‐temperature direct ethanol fuel cells (DEFCs) in the future, particularly for industrial‐scale applications.

## Author Contributions

**Sayyar Muhammad**: Supervision, Project administration, Conceptualization, Methodology, Writing original draft, **Najia**: Investigation, Resources, Formal analysis, Data curation, Validation, **Zarshad Khan**: Investigation, Formal analysis, Data curation, **Samina Aziz**: Investigation, Data curation, Formal analysis, **Hammad Khan**: Investigation, Formal analysis, Data curation, **Umair Hassan**: Formal analysis, Data curation, Investigation, **Mahrosh Iqbal**: Formal analysis, and Data curation **Jalal Khan**: Project administration, Methodology, Investigation, Writing, Formal analysis, **Asad Ali**: Conceptualization, Resources, Funding acquisition, Editing of draft.

## Conflict of Interests

The authors state that they have no known competing financial interests or personal relationships that may have influenced the presented work.

1

## Supporting information

As a service to our authors and readers, this journal provides supporting information supplied by the authors. Such materials are peer reviewed and may be re‐organized for online delivery, but are not copy‐edited or typeset. Technical support issues arising from supporting information (other than missing files) should be addressed to the authors.

Supporting Information

## Data Availability

The data that support the findings of this study are available from the corresponding author upon reasonable request.
