# Supplementary material for: Moisture Sorption by Low‐Cost Pyridinium‐Based Protic Ionic Liquids: Kinetics and Physico‐Electrochemical Properties
Source: ChemistryOpen. 2024 Nov 26;14(1):e202400165. doi: 10.1002/open.202400165 (PMC11726693; doi:10.1002/open.202400165)
Supplement: Supplementary file 1 — Supporting Information [file OPEN-14-e202400165-s001.pdf]

ChemistryOpen

Supporting Information

Moisture Sorption by Low-Cost Pyridinium-Based Protic Ionic Liquids: Kinetics and Physico-Electrochemical Properties

Sayyar Muhammad,* Najia, Zarshad Ali, Samina Aziz, Muhammad Hammad Khan, Maroosh Iqbal, Umair Hassan, Jalal Khan, and Asad Ali*

Supporting Information

Moisture Sorption by Low-Cost Pyridinium-Based Protic Ionic Liquids: Kinetics and Physico-Electrochemical Properties

Sayyar Muhammad [1,] Najia, [1] Zarshad Ali, [1] Samina Aziz, [1] Muhammad Hammad Khan, [1] Maroosh Iqbal, [1] Umair Hassan, [1] Jalal Khan, [1]Asad Ali[2 *]*

[1]Department of Chemistry, Islamia College Peshawar 25120-Peshawar Khyber Pakhtunkhwa, Pakistan

[2] Energy engineering, Division of Energy Science, Luleå University of Technology, 97187 Luleå Sweden

** Corresponding author: E-mail: sayyar@icp.edu.pk (S. Muhammad)*

Email: asad.ali@associated.ltu.se (A. Ali)

Figure S1: 1H-NMR spectra of [HPyr][HSO_4] taken with a 300MHz NMR instrument Bruker Perkin Elmer (UK) in deuterated chloroform ($CDCl_3$).

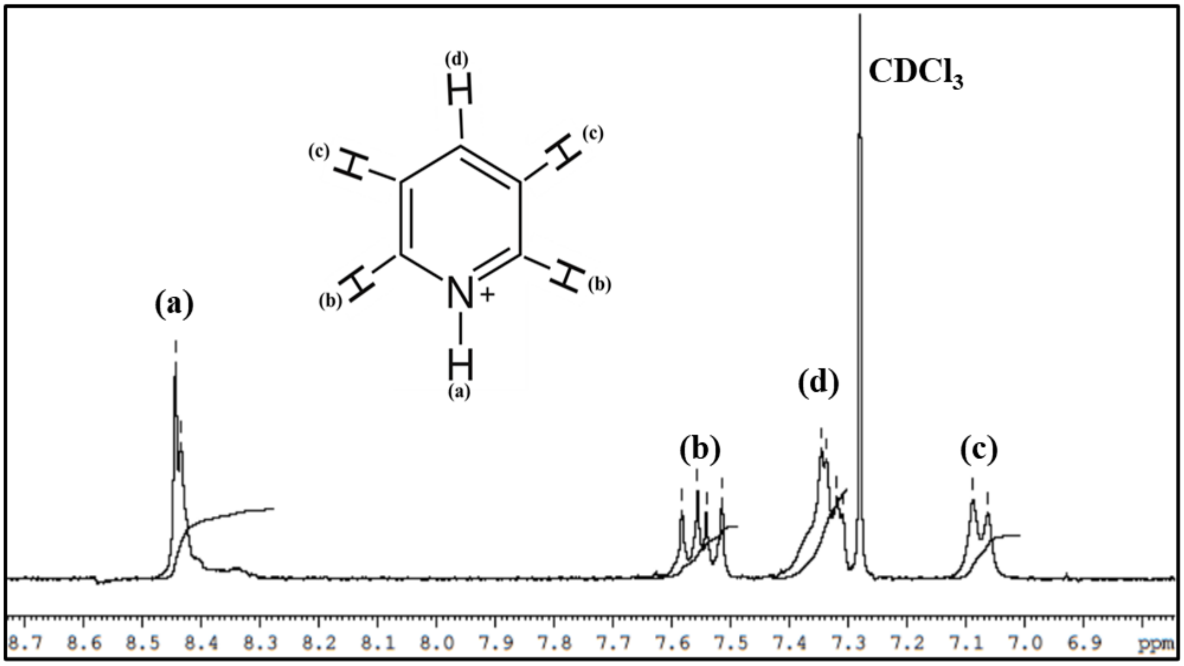

Assignment	Chemical Shift (ppm)
(a) 1H, duplet	8.444, 8.434
(b) 2H, quadruplet	7.583, 7.556, 7.542, 7.515

(d) 2H, quadruplet	7.346, 7.338, 7.319, 7.310
(c) 1H, duplet	7.089, 7.063

Table S1: shows %water contents and densities (g/mL) of [HPyr][HSO_4] and [HPyr]$_2$[SO_4] exposed to air for different time intervals.

**Time (Hrs.)**	**[HPyr][HSO_4]**			**[HPyr]$_2$[SO_4]**		
	**%H_2O**	**−ln% H_2O**	**Density**	**%H_2O**	**−ln% H_2O**	**Density**
**0**	0.765	0.27	1.690	0.661	0.42	1.380
**24**	0.810	0.21	1.670	0.698	0.36	1.371
**48**	0.828	0.19	1.640	0.786	0.24	1.361
**72**	0.835	0.18	1.607	0.801	0.22	1.334
**96**	0.862	0.15	1.521	0.822	0.20	1.314
**120**	0.895	0.11	1.481	0.849	0.16	1.301
**144**	0.951	0.05	1.447	0.867	0.14	1.298
**168**	0.987	0.01	1.358	0.894	0.11	1.285
**192**	1.01	-0.01	1.321	0.936	0.07	1.271
**216**	1.027	-0.03	1.302	0.961	0.04	1.236

Table S2: Conductivity, σ and logarithm of conductivity (ln σ) data of [HPyr][HSO_4] and [HPyr]$_2$[SO_4] at different temperatures.

**T (K)**	**1000/T (K^{-1})**	**[HPyr][HSO_4]**		**[HPyr]$_2$[SO_4]**	
		**Σ**	**ln σ**	**σ**	**ln σ**
**298**	3.356	110	4.62	114	4.74
**313**	3.195	128	4.85	136	4.91
**323**	3.096	147	4.99	155	5.04

**333**	3.003	175	5.16	186	5.23
**343**	2.915	194	5.27	206	5.33
**353**	2.833	221	5.40	242	5.49
**363**	2.755	253	5.53	279	5.63
**373**	2.681	290	5.67	325	5.78
**383**	2.611	320	5.77	345	5.84
**393**	2.545	334	5.81	356	5.93

Table S3: EWs of the PILs at Pt, Au, and GC working electrodes *vs.* Ag quasi reference at 50 mV/s and 303 K.

PIL's Name	Electrode	Anodic Limit (V)	Cathodic Limit (V)	EW (V)
**[HPyr][HSO_4]**	Pt	+2.0	-0.1	2.1
	Au	+1.8	-0.5	2.3
**[HPyr]$_2$[SO_4]**	Pt	+2.2	-0.1	2.3
	Au	+1.9	-0.5	2.4

Figure S2: CVs taken in [HPyr][HSO_4] and [HPyr]$_2$[SO_4] at a scan rate of 50 mV/s at Pt and Au electrodes showing the effect of temperature on the EW of the PILs.

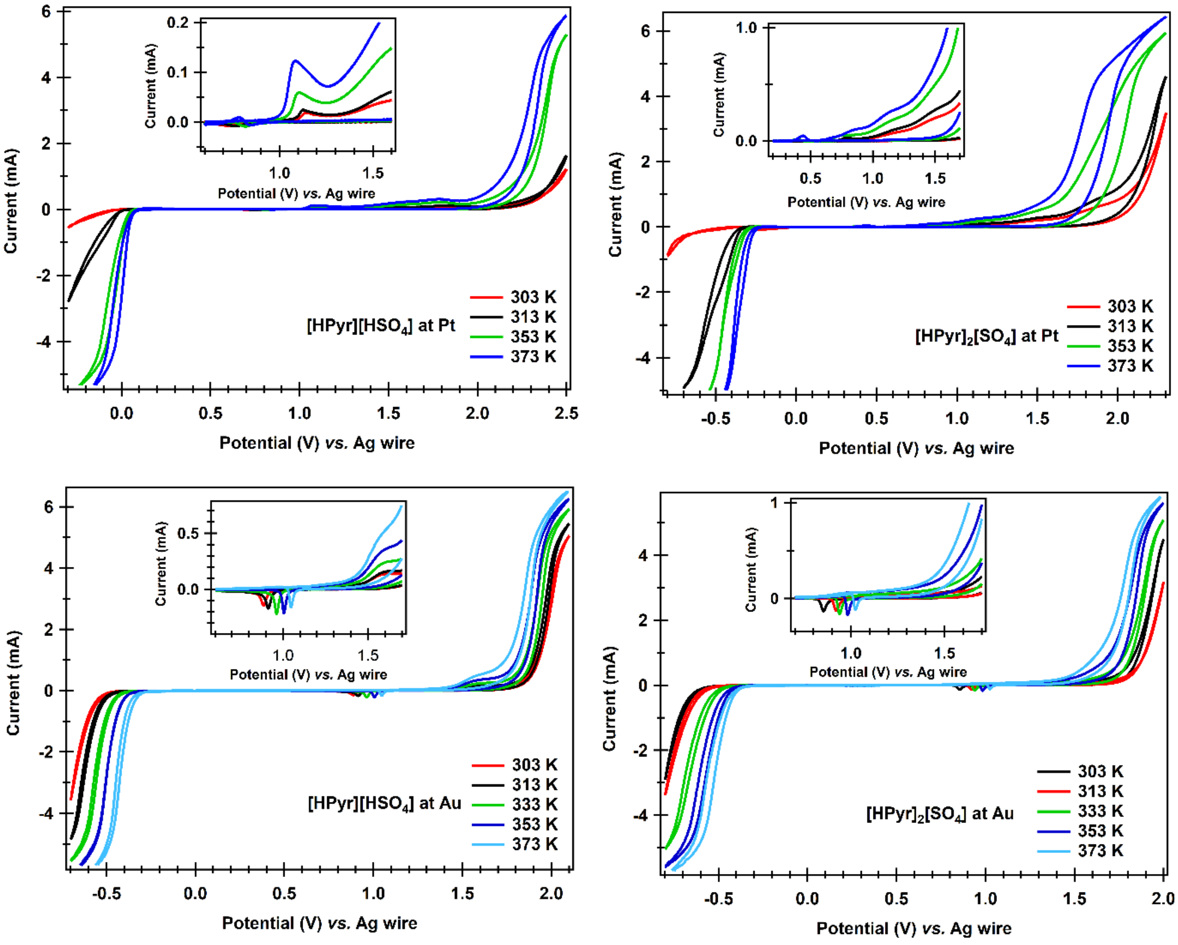
[HPyr][HSO4] at Pt
[HPyr]2[SO4] at Pt
[HPyr][HSO4] at Au
[HPyr]2[SO4] at Au
303 K
313 K
333 K
353 K
373 K
Current (mA)
Potential (V) vs. Ag wire
